# A coordinated switch in sucrose and callose metabolism enables enhanced symplastic unloading in potato tubers

**DOI:** 10.1017/qpb.2024.4

**Published:** 2024-04-17

**Authors:** Bas van den Herik, Sara Bergonzi, Yingji Li, Christian W. Bachem, Kirsten H. ten Tusscher

**Affiliations:** 1 Computational Developmental Biology, Utrecht University, Utrecht, The Netherlands; 2 Plant Breeding, Wageningen University & Research, Wageningen, The Netherlands

**Keywords:** Callose, Sucrose, unloading, potato

## Abstract

One of the early changes upon tuber induction is the switch from apoplastic to symplastic unloading. Whether and how this change in unloading mode contributes to sink strength has remained unclear. In addition, developing tubers also change from energy to storage-based sucrose metabolism. Here, we investigated the coordination between changes in unloading mode and sucrose metabolism and their relative role in tuber sink strength by looking into callose and sucrose metabolism gene expression combined with a model of apoplastic and symplastic unloading. Gene expression analysis suggests that callose deposition in tubers is decreased by lower callose synthase expression. Furthermore, changes in callose and sucrose metabolism are strongly correlated, indicating a well-coordinated developmental switch. Modelling indicates that symplastic unloading is not the most efficient unloading mode per se. Instead, it is the concurrent metabolic switch that provides the physiological conditions necessary to potentiate symplastic transport and thereby enhance tuber sink strength .

## 1. Introduction

In potato, the tuberigen *StSP6A* induces tuber onset (Navarro et al., 2011), which is a major developmental transition, associated with large changes in plant physiology among which is the emergence of a new and strong sucrose sink. Besides its role in tuber establishment by switching on the tuber developmental programme, *StSP6A* was shown to inhibit sucrose export from the phloem to the apoplast through the inhibition of SWEET transporters (Abelenda et al., 2019), thereby enhancing the efficiency of sucrose delivery to sink tissues (van den Herik et al., 2021). Using a biophysical model of sugar and water transport, we recently demonstrated that this dual role of *StSP6A*, tuber induction and inhibition of SWEET-mediated export, preferentially enhances sucrose allocation to the tuber sink (van den Herik & ten Tusscher, 2022). A remaining open question is what processes make tubers strong sinks, and to what extent *StSP6A* is involved in controlling these processes.

While *StSP6A*-mediated induction of tuberization is a logical first step in establishing a new strong sink, whether and how the switch from apoplastic to symplastic unloading (Viola et al., 2001) contributes to sink strength has remained unclear. Although symplastic unloading is generally believed to enhance unloading efficiency and thereby contribute to sink strength (Fernie et al., 2020; Viola et al., 2001), this is largely based on data comparing different species and tissues rather than comparing the two distinct unloading modes for a single tissue. Additionally, the processes guiding this unloading switch and the potential role of *StSP6A* therein have not yet been elucidated. In addition to inhibition of the apoplastic route through *StSP6A* inhibition of SWEETs, a coordinated promotion of the symplastic route is likely to occur.

Plasmodesmal aperture, a key factor determining the efficiency of symplastic transport, is regulated by callose deposition at the neck of the plasmodesmata, with reduced callose deposition opening plasmodesmata. Callose homeostasis is regulated by two antagonistic gene families, callose synthases (CalS), producing callose and β-1,3-glucanase (1,3-BG)-degrading callose (Amsbury et al., 2018; De Storme & Geelen, 2014; Wu et al., 2018). We therefore investigate whether upon tuberization onset changes in callose levels and the expression of callose homeostasis genes occur.

Another transition occurring during tuber formation is the switch from energy to storage metabolism. This switch involves a transition from apoplastic cell wall invertase (cwInv) to cytoplasmic sucrose synthase (SuSy)-mediated sucrose cleavage (Viola et al., 2001). While the former delivers glucose and fructose for energy metabolism, the latter serves as an initial step in the formation of starch for storage by directly yielding UDP-glucose, the precursor for starch synthesis (Nazarian-Firouzabadi & Visser, 2017). Potato tubers have been shown to depend on a switch to SuSy usage during tuber growth to prevent inefficient growth and storage (Bologa et al., 2003). During the early stages of tuber growth, there is a substantial increase in the hexose/sucrose ratio, and a decrease in total sugars occurs. The decrease in total sugars is indicative of an increased starch synthesis rate (Oparka et al., 1990), whereas a decreased hexose/sucrose ratio is likely due to increased metabolism rates of hexoses derived from sucrose, coupled with an increased flux of sucrose into the developing tip (Davies, 1984; Ross & Davies, 1992). Altogether, there is thus enzymatic and metabolic evidence for a functional enzymatic switch to occur. Still, an open question is whether this enzymatic switch is triggered by the change in sucrose delivery due to the apoplastic to symplastic switch, or rather is part of a more coordinated developmental programme changing both transport mode and metabolism. We therefore investigate the extent of concurrence of the changes in callose and sucrose metabolism.

The switch to the formation and storage of non-soluble starch and the concurrent decrease in soluble sugars may enhance sink strength under symplastic unloading by maintaining a concentration gradient towards the sink. Alternatively, the increased sink strength following the unloading switch may involve the differences between the transport modes themselves. Passive symplastic transport through plasmodesmata is generally regarded one or a few orders of magnitude more efficient than active apoplastic transport (Patrick & Offler, 1996). Moreover, symplastic transport reduces the energy spent on sucrose delivery by eliminating the energy required to maintain the proton motive force and reduces the requirement for significant investment in vascular tissue (Patrick & Offler, 1996). The relative efficiency of the two modes of transport will thus likely depend on the physiological conditions present in the stolon and tuber, and enhanced sink strength may involve both transport mode and metabolism. To investigate the coupling between transport mode and metabolism, we used a biophysical model to compare apoplastic versus symplastic unloading efficiency as a function of transporter and plasmodesmata densities, local sucrose concentration and concentration gradients.

## 2. Materials and methods

### 2.1. In vitro plant growth and microscopy

*Solanum andigena* was propagated *in vitro* on MS20 medium (MS, 20 g/L saccharose, pH 5.8), and cultivated at 24°C for 4 weeks. The nodes from the upper three nodal stem sections were selected with each node containing an expanded leaf and a single axillary bud. At least 40 decapitated single-node cuttings were cut and propagated in MS20 medium under dark conditions at 20°C for 5–10 days. Explants with elongated stolons were moved to tuber induction medium (MS, 80 g/L saccharose, 1.5 mL/L 6-benzylaminopurin, pH 5.8) and cultivated in similar conditions as stolon elongation.

For microscopy, five samples were harvested at three different development stages; non-swelling stolon (stage 1), swelling stolon/small tuber (stage 2 or stage 3) and large tuber (stage 4), as described by Viola et al. (2001). Harvested stolon and tuber samples were cut using a hand microtome. Samples were then stained in a 150 mM K_2_PO_4_ (PH = 9) and 0.01% aniline blue solution for 2 h in the dark. Callose deposition was imaged with a DAPI filter (4FL) and an excitation wavelength of 370 nm.

### 2.2. Sequence retrieval, phylogenetic analysis and functional annotation

Protein sequences of the 1,3-BG, CalS and invertase families were identified using the Phytozome database (Goodstein et al., 2012) and a PSI-BLAST (Altschul et al., 1997) search was performed for each family to identify similar sequences missing or wrongly annotated in the Phytozome database. We performed the same search for the already classified SuSy (11 members), SWEET (35 members) and SUT (3 members) gene families, but no unknown genes were found using this approach. For the SUT family, which is larger in Arabidopsis with nine members (Niño-González et al., 2019), we decided to specifically focus on the SUT subfamily, and to not include the larger MFS family as we are investigating sucrose transport over the plasma membrane. For the 1,3-BG, CalS and invertase families, multiple sequence alignments were made with the auto option in MAFFT v7.310 (Katoh & Standley, 2013) and trimmed with trimAl v1.rev15 (Capella-Gutiérrez et al., 2009) using a gap threshold of 10%. Phylogenetic trees were constructed with IQ-TREE v1.5.5 (Nguyen et al., 2015) using ModelFinder (Kalyaanamoorthy et al., 2017) and ultrafast bootstrap approximation (Hoang et al., 2018).

The 1,3BG family was functionally annotated for features previously associated with the protein family, similarly to the approach used by Paniagua et al. (2021); signal peptide (SP), glycosylphosphatidylinositol (GPI) anchors, and X8 (CBM43) domains were identified. Prediction of these features was done using SMART (Schultz et al., 2000) and Interpro (Mitchell et al., 2019). SPs were further predicted using SignalP v5.0 (Almagro Armenteros et al., 2019), and presence of GPI anchor was further predicted using PredGPI (Pierleoni et al., 2008). Sequence signatures were considered present when predicted by two or more databases.

### 2.3. In silico gene expression analysis

Microarray expression data of 24 tissue and organ samples from *Solanum tuberosum* Group Phureja DM 1-3 516 R44 (DM) were obtained from spudDB (Pham et al., 2020). These samples were a selection of all samples without applied stress and/or treatment free. Similarly, 15 samples from the *S. tuberosum* group Tuberosum RH89-039-16 (RH) (Zhou et al., 2020) were analysed to confirm the observations in DM. The DM dataset contained two stolon and three tuber samples that were investigated in more detail. Analysis of the expression data was performed in python using the clustermap function from the seaborn package (Waskom, 2021), expression was normalized using Z-score normalization and hierarchical clustering was performed using the ‘ward’ method implemented in seaborn.

### 2.4. Unloading model

Unloading rates for three different unloading modes are compared for varying sucrose concentrations in the phloem (*C*_phloem_) and parenchyma (*C*_parenchyma_). Below, we describe the models and parameter derivation, all parameter values are given in Table 1. Apoplastic or active unloading (*I*_a_) is described by a Michaelis–Menten term dependent on the phloem sucrose concentration (Eq. (1)):
(1)

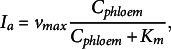

**Table 1:** Variables and parameters for the potato sucrose unloading model.

Parameter/variable	Symbol	Value (range)	Units	Reference
Sucrose concentration phloem	*C*_Phloem_	Variable	mol m^–3^	–
Sucrose concentration parenchyma	*C*_Parenchyma_	Variable	mol m^–3^	–
Maximum transport rate SWEET transporter	*v*_max_	0.93 (0.5–2)	10^–19^mol μm^–2^ s^–1^	Chen et al., (2012)
Michaelis–Menten constant SWEET transporter	*K*_M_	50 (10–75)	mol m^–3^	Chen et al., (2012)
Plasmodesmata density	*N*	4 (3–6)	μm^–1^	Oparka, (1986)
Diffusion constant sucrose	*D*	5e–10	m^2^ s^–1^	–
Cell wall thickness	*l*	1 (0.3–1.2)	μm	Gibson, (2012); Reeve et al., (1973)
Plasmodesmata outer radius	*r*_outer_	10	nm	Ross–Elliot et al., 2017
Desmotubule radius	*r*_dt_	8	nm	Ross–Elliot et al., 2017
Cystoplasmic sleeve width	*w*	2	nm	Ross–Elliot et al., 2017
Gas constant	*R*	8.31	Pa m^3^ K^–1^ mol^–1^	–
Temperature	*T*	293	K	–
Partial molal volume sucrose	*V*_suc_	0.22	dm^3^mol^–1^	–
Dynamic viscosity of water	η_0_	1.002	mPa s	–

*Note:* Values in brackets represent the reported range of the parameter in potato.

where for *v*_max_ and *K*_m_, SWEET specific parameters are taken. We thus explicitly model SWEET-mediated sucrose export but not SUT-mediated sucrose import. Implicit in this model formulation is the assumption that in active phloem unloading from the sieve-element/companion-cell complex to the tuber parenchyma SWEET-transporter kinetics are the rate limiting step. Supporting this assumption, SWEET and SUT-transporters have comparable maximum transport rates (van den Herik et al., 2021) while SUTs have a substantially higher sucrose affinity than SWEETS (i.e., lower *K*_m_ of 1 mM for SUT compared to 10–75 mM for SWEETS; Chen et al., 2012; Riesmeier et al., 1993). As a consequence, SWEETS are more likely to operate at non-maximum transport rates than SUTs, rendering them the rate limiting factor. SWEET rates were reported as 39 ± 6 pmol/oocyte/min (Chen et al., 2012), which was rewritten to 0.9e–19 mol/μm^2^/s by using reported oocyte dimensions (Wallace & Selman, 1981). Reported SWEET *K*_m_ values range from 10 to 75 mM (Chen et al., 2012), here an intermediate value of 50 mM was used.

To simulate symplastic transport, we used the model for simple plasmodesmata developed by Ross-Elliott et al. (2017). This model described two symplastic unloading modes; diffusive symplastic transport and symplastic bulk flow, both through plasmodesmata. Both models therefore need plasmodesmal area as important input. Individual plasmodesmal area is calculated by subtracting the surface area of the desmotubule from the total area, assuming that both the plasmodesmal and desmotubule surface area is circular. Total plasmodesmal area (*A*_PD_) is then calculated by multiplying individual area with the total number of plasmodesmata per μm^2^:
(2)

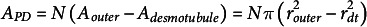



Diffusive unloading through plasmodesmata (*I*_D_) is described as standard diffusion through a simple collection of pores (Eq. (3)) with area *A*_PD_ and pore length *l*. Diffusion thus relies on the sugar concentration gradient between the phloem and parenchyma ( 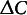 ):
(3)

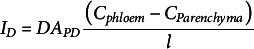



Bulk flow through plasmodesmata (*I*_b_) is described by the volumetric water flow through the plasmodesmata (*Q*) as well as the sugar concentration in the phloem (Eq. (4)):
(4)

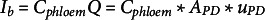



The volumetric water flow through plasmodesmata is driven by the pressure differential between the phloem and parenchyma, we here assume that this pressure differential is solely dependent on the osmotic pressure differential, and as such that turgor pressure in the phloem and surrounding parenchyma is equal (Eq. (5)):
(5)

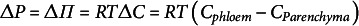



With *R* being the universal gas constant and *T* the absolute temperature.

Water flow velocity through plasmodesmata is estimated as flow through a straight slit of width *w* (where *w* = *r*_outer_ – *r*_dt_) and length l as described in detail by Ross-Elliot et al. (2017), resulting in Eq. (6):
(6)

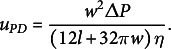



In this model version, the viscosity of the solution flowing through the plasmodesmata (η) was solute dependent (Eq. (7)). Plasmodesmal sugar concentration was estimated as the average between phloem and parenchyma:
(7)

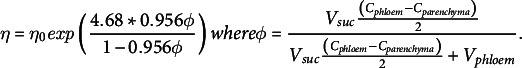



Thus, bulk transport is linearly dependent on phloem sucrose concentration (Eq. (4)), linearly dependent on the phloem parenchyma sucrose concentration difference determining the osmotic pressure differential (Eq. (5)), and non-linearly dependent on this same concentration difference via the viscosity of the phloem sap (Eq. (7)).

## 3. Results

### 3.1. Starch presence interferes with callose deposition quantification in tubers

**Figure 1.**
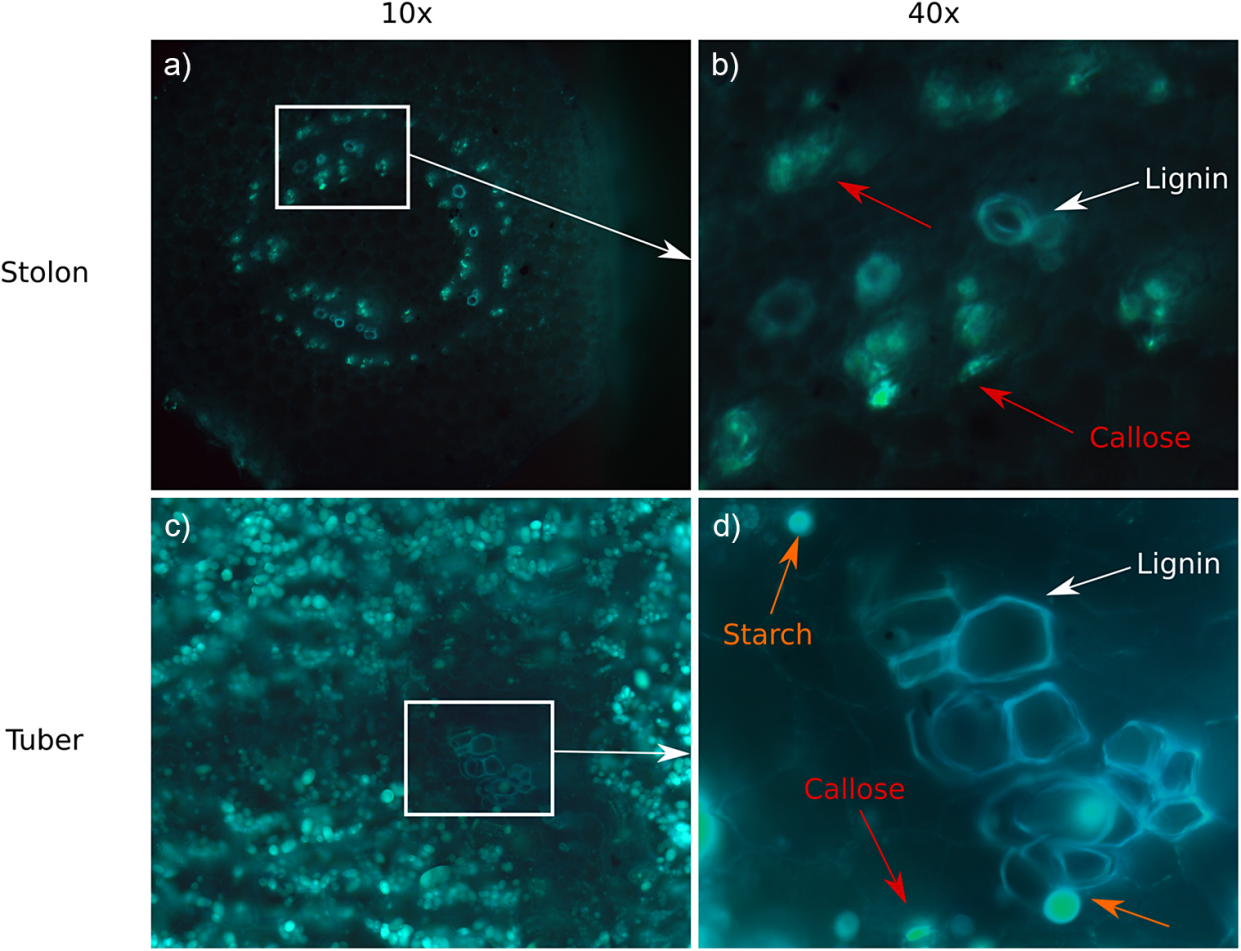
Callose presence at stolons and tubers. (a) Longitudinal sample of a non-swelling stolon at 10x magnification. (b) 40x magnification of part of the sample in panel (a). Callose (red arrows) and lignin (white arrows) are clearly visible in the phloem and xylem. (c) Longitudinal sample of the vasculature of a large tuber (stage 4) at 10x magnification. (d) 40x magnification of part of the sample in panel (c). Callose (red arrows), lignin (white arrows) and starch (orange arrows) are visible in the phloem and xylem.

To investigate whether callose levels decrease during tuber formation, we used fluorescence microscopy, using aniline blue to stain callose. Callose could be observed in both stolon and tuber samples, with clear presence of callose in both phloem rings in the stolon (Figure 1a,b). Xylem vessels were also clearly visible due to the fluorescent properties of lignin (Albinsson et al., 1999). Callose could be distinguished from lignin due to differences in coloration, with lignin appearing bluer than callose under our experimental conditions. Unfortunately, callose and starch displayed a similar green colour, although starch depositions appear to have a more rounded, granular appearance and callose having a more ring like shape. As a consequence, while callose abundance appeared to have decreased in the vascular region of tuber samples (Figure 1c,d), precise, unambiguous quantification of callose levels in tubers versus stolons was prohibited by the large amount of starch in tubers. Thus, we could not conclude with certainty that callose levels decrease upon the transition to tuberization based on this method. Therefore, to obtain further support for a suspected decline in callose levels to enable the known switch to symplastic unloading, we decided to investigate sugar and callose metabolism dynamics at the expression level.

### 3.2. Sequence retrieval and phylogeny of the 1,3-BG, CalS and invertase gene families

To investigate gene expression patterns, a classification of sugar and callose metabolism gene families was needed. While the SuSy (Van Harsselaar et al., 2017; Xu et al., 2019), SWEET (Manck-Götzenberger & Requena, 2016) and SUT (Chen et al., 2022; Chincinska et al., 2008) families were previously classified (Table 2) and an additional search for unknown gene family members did not result in new genes for these families (see the section “Materials and methods”). An overview of potato 1,3-BG, CalS and sucrose invertase genes was lacking. We first identified the genes constituting these families and performed a phylogenetic and functional analysis to get an overview of the number of genes (Table 2) present and their expected functional role in tuber development.

A total of 62 1,3-BG genes encoding callose-degrading enzymes were identified using the phytozome database combined with a psi-BLAST search. Together with a group of eight *Arabidopsis thaliana* 1,3-BG genes previously identified to be plasmodesmata-associated (Levy et al., 2007; Wu et al., 2018) containing one or more representatives of each of the three previously identified clades (α,β,γ) (Doxey et al., 2007), a phylogenetic tree was constructed (Figure S1 in the Supplementary Material). Genes were functionally annotated for the presence of an SP (excretion), GPI anchor (membrane anchorage) and X8 domain (carbohydrate binding/plasmodesmata association). Our results indicate that the three previously described clades are all present in potato and differ significantly in the presence/absence of functional domains. Specifically, the α-clade is very diverse with regard to the presence of the domains, while the β-clade is characterized by presence of an excretion SP, GPI anchor, and X8 domain in most proteins (13 out of 17), and the γ-clade is characterized by a complete absence of GPI anchor and X8 domain, similar to what is found in Arabidopsis (Doxey et al., 2007) and tomato (Paniagua et al., 2021). Based on this, proteins active in callose degradation at the PD are expected to be mainly localized in the α- or β-clade. Indeed, all 1,3-BG characterized to be PD-related in Arabidopsis are located in the α-clade (Levy et al., 2007).

A total of 13 CalS genes encoding callose producing enzymes were identified, 6 sequences were discarded as they lacked the UDP-glucose catalytic site needed for enzymatic activity (Hong et al., 2001). The 7 remaining CalS sequences, together with the 13 CalS genes reported in Arabidopsis (Richmond & Somerville, 2000) were used to construct a phylogenetic tree (Figure S2 in the Supplementary Material). The tree was annotated with known functions for the Arabidopsis genes (Wu et al., 2018), inferring similar roles for potato genes from their Arabidopsis orthologs. Based on this analysis, two putative PD-associated proteins were identified (Soltu.DM.07G023050 and Soltu.DM.01G001920).

Finally, with regard to invertases involved in sucrose degradation, three main clades exist, acidic cwInv, neutral/alkylic soluble invertase (Inv) and vacuolar invertases (vInv). These three classes have largely different physiological roles (Roitsch & González, 2004). A total of 25 invertase genes were identified in potato. Not all genes were functionally annotated, and function was therefore inferred using their phylogeny and partial annotation in potato (Figure S3 in the Supplementary Material), based on this analysis 9 cwInv, 11 Inv and 5 vInv are present.

**Table 2:** Overview of the callose and sucrose gene families.

Gene family	Potato members	Reference
β-1,3–glucanase	62	This work
Callose synthase	7	This work
Invertase	25 (9 cwInv, 11 Inv, 5 vInv)	This work
Sucrose synthase	11	Van Harsselaar et al., (2017); Xu et al., (2019)
SWEET	35	Manck–Gotzenberger & Requena, (2016)
SUT	3	Chincinska et al., (2008); Chen et al., (2022)

### 3.3. The highly diverse 1,3-BG and CalS families show clear developmental expression clusters

We next investigated the expression patterns of the 1,3-BG and CalS families to investigate changes in callose homeostasis during tuberization. For this, we made use of a dataset containing microarray gene expression data across a variety of organs of *S. tuberosum* Group Phureja DM1-3 (DM) (Pham et al, 2020). Distinct developmental expression clusters are present, with flowers and stamens, petioles, fruit, stolons and tubers all clustering separately (Figure S4 in the Supplementary Material). Stolons did show partially similar expression patterns to shoot and root samples, which is expected from the shared stem-like nature of these three organ types. The observed clusters reflect the functional diversity of callose besides its role in regulation of the plasmodesmal aperture, as callose also plays a role in cell wall integrity and mechanics, response to (a)biotic stresses, pollen development and cellular differentiation (Amsbury et al., 2018; Levy et al., 2007; Wu et al., 2018).

**Figure 2.**
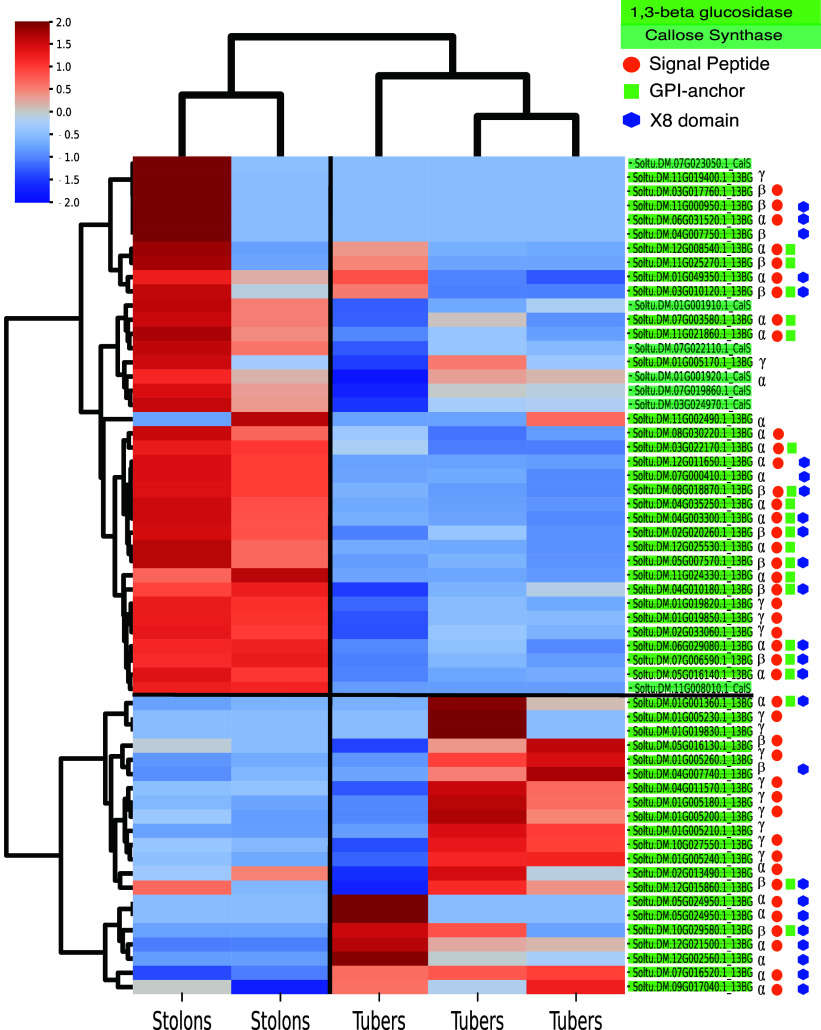
1,3-BG and CalS expression in stolon and tuber samples. *Z*-score normalized rows with hierarchical clustering shows distinct stolon and tuber clusters. Clade and annotation of SPs, GPI anchors and X8 domains is visualized behind each 1,3-BG gene.

Subsequently, three tuber and two stolon tissue samples available in the DM dataset were investigated in more detail. In these five samples, a subset of 52 of the 62 1,3-BG genes and all 7 identified CalS genes were expressed (Figure 2). Stolon samples clustered together, with all CalS genes showing expression in stolon samples and none showing high expression in the tubers. Tuber samples were also strongly correlated, with two showing very strong overlap in 1,3-BG expression and the third sample having a separate set of 1,3-BGs expressed. Tuber samples mainly expressed 1,3-BGs from the γ-clade, lacking both a GPI anchor as well as a X8 domain, with only 2 out of the 21 1,3-BG genes having a GPI anchor (Figure 2). In contrast, in stolon expression of α- and β-clade is dominant and 17 out of 31 1,3-BG genes contained a GPI-anchor domain. The majority of 1,3-BGs expressed in stolons are thus membrane- and/or PD-associated, suggesting a role in callose degradation. In contrast, in tubers, expressed genes are associated with pathogen resistance and cell wall remodelling (Doxey et al., 2007), likely due to their secretory nature. One of the three tuber samples showed a significantly different expression pattern for 1,3-BG genes than the other two. Importantly, despite these differences with regard to individual genes, all three samples consistently showed mainly 1,3-BG expression and no CalS expression in tubers as compared to stolons. To further verify these observations, the same analysis was performed on a stolon and tuber expression dataset of *S. tuberosum* Group Tuberosum RH89-039-16 (RH) (Zhou et al., 2020), providing similar results (Figure S5 in the Supplementary Material). Combined, this shows that CalS is mainly expressed in stolons, whereas 1,3-BG is expressed in both stolons and tubers, albeit that functionally different genes are expressed. Importantly, while CalS expression in the stolons does not proof activity, the absence of expression in tubers does indicate the absence of callose production there. Furthermore, the switching off of CalS upon the developmental transition to tuber formation does support that their prior expression in stolons was functionally relevant. Combined this suggests a major switch in callose homeostasis upon tuber formation.

### 3.4. A clear developmental pattern is present for callose and sucrose metabolism

**Figure 3.**
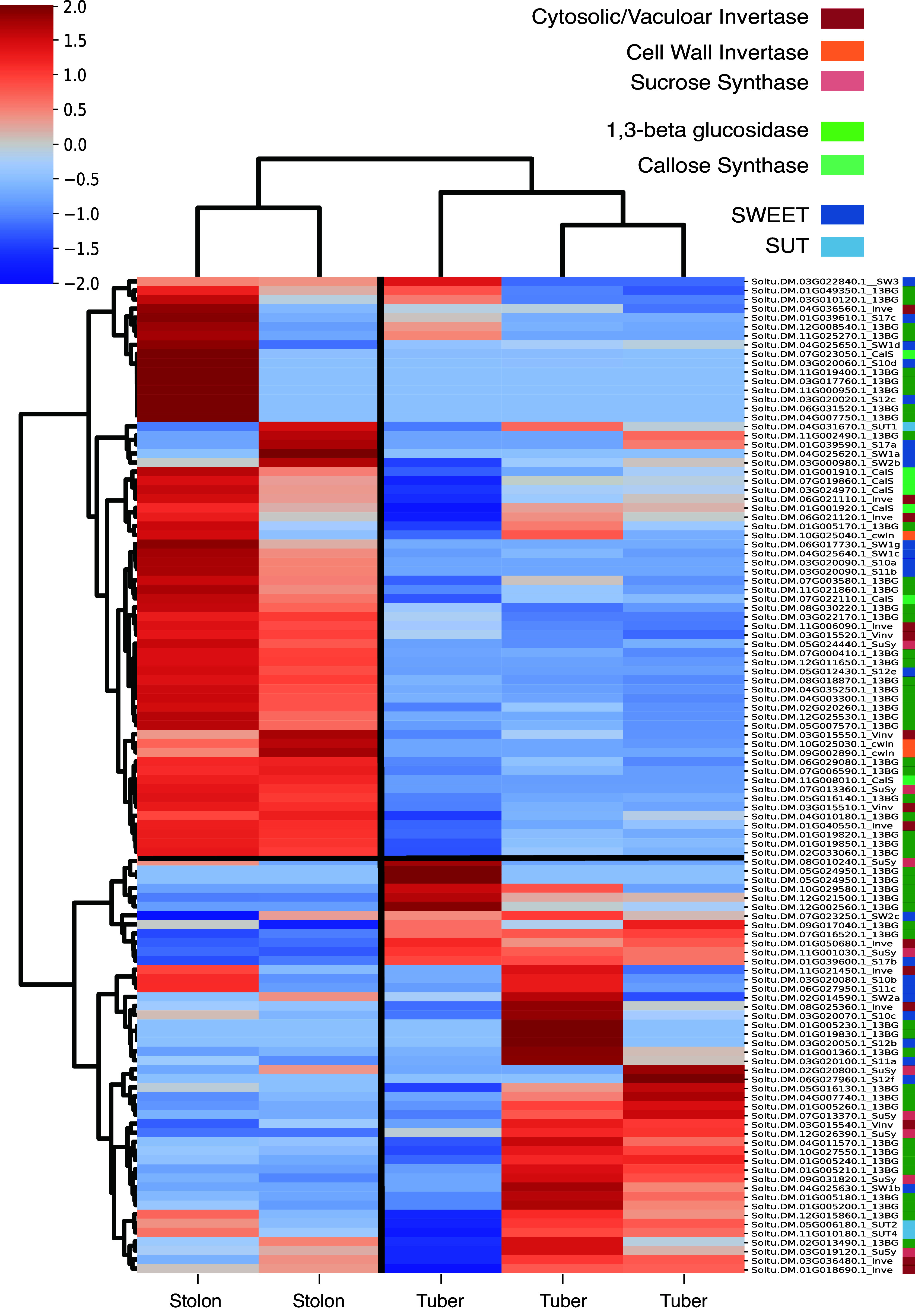
Heatmap of expression profiles of sugar metabolism, transport and callose balancing in stolon and tuber samples of DM. *Z*-score normalized rows with hierarchical clustering show similar stolon and tuber clusters as observed in Figure 2. Colours behind the genes depict gene family.

Besides changes in callose homeostasis, a rewiring of sucrose metabolism as well as changes in sugar transporters are known to occur during tuberization (Jing et al., 2023; Viola et al., 2001). To investigate if and how these processes are coupled during tuberization the gene expression of callose (1,3-BG and CalS) and sucrose metabolism (SuSy, cwInv, vInv and Inv) as well as sucrose transporters (SWEET and SUT) were investigated alone, as well as in combination (Figures S3 and S6 in the Supplementary Material). Clearly, distinct stolon and tuber clusters were observed when considering sucrose metabolism in isolation (Figure S6 in the Supplementary Material), as well as combined with sucrose transporters and callose metabolism (Figure 3).

During the developmental switch from stolon to tuber both cytoplasmic and cwInv activity is reported to decrease, whereas SuSy activity increases (Viola et al., 2001). Three out of nine cwInv are expressed in the stolon cluster and show no to very low expression levels in tubers (Figures S4 and S6 in the Supplementary Material). The other six cwInv were not expressed in either stolons or tubers. Soluble and vacuolar invertases showed more mixed expression patterns, with five expressed in the stolon cluster, and six expressed in the tuber cluster. Nine SuSy genes showed expression in stolons and tubers, with the majority expressed in tubers (seven out of nine). SWEET and SUT expression patterns were more ambiguous than sucrose and callose metabolism, consistent with earlier observations (Jing et al., 2023), and a more important role for post-translational interactions such as between *StSP6A* and *StSWEET11b* is expected for these families.

### 3.5. Enzymatic changes create favoured conditions for passive unloading

**Figure 4.**
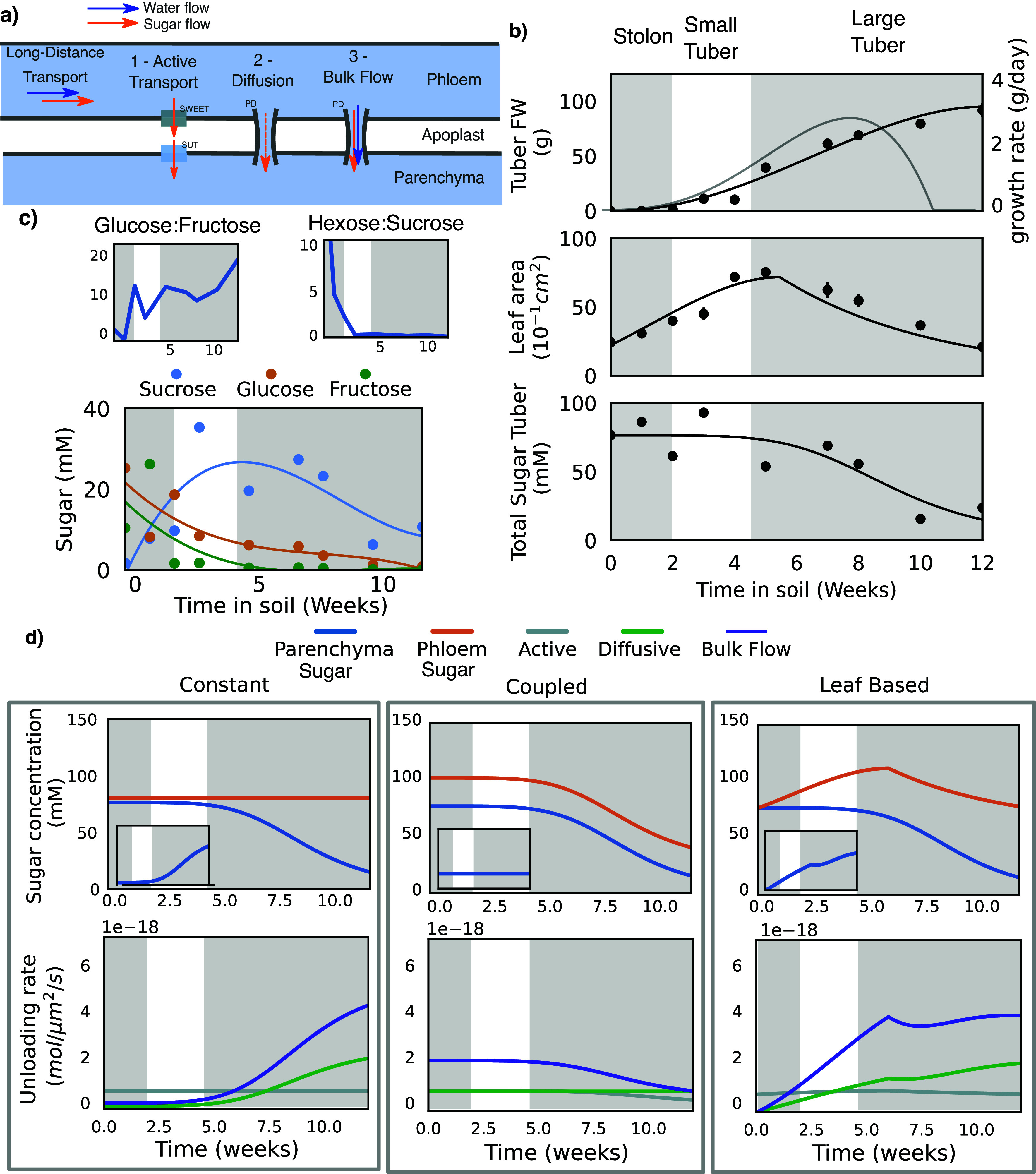
Sucrose unloading under different physiological conditions in stolon and tuber. (a) Schematic overview of the unloading model. (b) Potato tuber fresh weight, leaf area and tuber sugar dynamics over plant development (van den Herik et al., 2023). The first grey box depicts the stolon stage, the white box depicts the small tuber stage and the second grey box the large tuber stage, as described by Viola et al. (2001). (c) Sucrose, fructose and glucose dynamics in stolons and tuber (van den Herik et al., 2023). Glucose:Fructose and Hexose:Sucrose ratios are calculated from the same data. (d) Model results for the three phloem concentration scenarios (columns). The top row shows the sucrose levels in the phloem and parenchyma (see panel (b)), with the inset showing the gradient between the phloem and sucrose. The bottom row gives the unloading rates for the three unloading modes as obtained from the simulations.

Above we demonstrated how the switch from stolon to tuber involves concurrent changes in both sucrose and callose metabol ism, both of which may contribute to the formation of a strong tuber sink. Here, we investigate the relative importance of sucrose physiology versus the unloading mode in determining sink strength. We first describe the physiological changes in terms of phloem and parenchyma sugar concentrations. Combined with the estimated sucrose transporter levels and plasmodesmata density, we parameterized a simple apoplastic and symplastic unloading model developed by Ross-Elliot et al. (2017) for the stolon and tuber (Figure 4a) (see the section “Materials and methods”).

Experimental data indicate that overall tuber sugar levels decrease during the exponential tuber growth phase (Figure 4b, bottom panel, van den Herik et al., 2023), consistent with the observed switch from energy to storage metabolism described above. Individual reducing sugars (glucose, fructose) and sucrose dynamics are more complex (Davies, 1984), with glucose and fructose levels already decreasing during stolon stages and sucrose only decreasing in the exponential tuber growth phase (Figure 4c). For our model, we assumed that the measured sugar concentrations in whole tuber samples are indicative of tuber parenchyma conditions, given the volumetric dominance of the storage parenchyma over the phloem locally delivering the sugar. In the absence of data on phloem sucrose levels in the stolon/tuber unloading zone, we investigated three different phloem sugar concentration scenarios: 1) constant concentration over tuber development (balanced source/sink dynamics: as demand increases supply increases accordingly, thus maintaining phloem concentration), 2) coupled phloem/parenchyma concentrations (local sink dynamics dominate: as demand increases supply cannot keep up and phloem concentration decreases) and 3) leaf-area-based concentration (source dynamics dominate: supply and hence phloem concentration is linearly related to leaf area). For all three scenarios, phloem concentrations in the tuber unloading zone were estimated based on model outcomes from a previously developed biophysical transport model in potato (Van den Herik et al, 2021). This model predicted mean unloading zone concentrations of the order of 100 mM, much lower than the sucrose concentration in the leaf and stem phloem, which can be up to 1.7 M in the model and was measured to be 1.35 M (Kehr et al., 1998) to 1.8 M (Pescod et al., 2007) in potato leaf phloem.

Due to the relatively low *K*_m_ of SWEET transporters compared to phloem concentrations (in the simulations we used an intermediate value of 50 mM as reported by Chen et al., 2012), apoplastic transport is close to saturation (due to the high concentration of 75–125 mM in the potato phloem) under most conditions, except for the coupled scenario, where lower concentrations during tuber bulking cause transport to decrease too half the maximum rate during development (Figure 4d, grey lines). Decreasing the *K*_m_ of the SWEET transporters to the lowest reported value of 10 mM does not change the model dynamics because of the already highly saturated regime in the model. SUT transporters have an even lower *K*_m_ of around 1 mM (Riesmeier et al., 1993), and thus operate close to saturation for all tested concentrations. Consequently, apoplastic transport is largely constant across concentrations and scenarios. In contrast, diffusive and bulk flow unloading show large differences over development and between the scenarios. As diffusion is only dependent on the concentration gradient between the phloem and parenchyma, the unloading rate parallels the concentration gradient (Figure 4d, insets and green lines). Plasmodesmal bulk flow depends on both the sugar concentration gradient, which drives osmotic pressure and hence flow rate, and the concentration of sucrose transported in the phloem. Therefore, bulk flow increases more strongly with the concentration gradient, resulting in a higher unloading potential than diffusion under all conditions, as was also observed in the developing phloem of Arabidopsis (Ross-Elliott et al., 2017).

Our model demonstrates that the switch to storage metabolism that results in a decrease in tuber sugar levels potentiates diffusive and bulk symplastic unloading in both the conservative, constant and data-based, leaf-surface-driven scenarios where sugar concentration gradients increase.

In both cases, bulk flow significantly exceeds diffusive transport due to the non-linear dependence of bulk flow on concentration levels via osmotic pressure and convective solute flow (Figure S7 in the Supplementary Material). In contrast, in the coupled scenario, where concentration gradients remain constant while phloem sucrose levels decrease, diffusive unloading efficiency remains constant while bulk unloading efficiency declines over development. This scenario is, however, deemed unlikely given the observed leaf growth dynamics, as well as the known negative feedback regulation of sucrose levels on photosynthesis, which likely prevents a fully parallel decline of phloem sucrose levels with sink levels.

Importantly, since we estimated transporter expression levels, plasmodesmal densities and apertures, model outcomes depend on precise parameter settings. To test the robustness of our results, we performed simulations with twofold higher transporter levels, twofold lower plasmodesmal densities and these changes combined (Figure S8 in the Supplementary Material). Our results show that while the precise rates of transport and the cross-over point from whereon symplastic transport is more efficient shifts, no qualitative changes occur. Put differently, we consistently observe that during early tuber formation apoplastic transport is more efficient, while during later stages symplastic transport dominates. The model thus clearly demonstrates that the physiological conditions created by the enzymatic switch (decreasing parenchyma concentration due to increasing starch synthesis) and general plant dynamics (leaf area increase) unlocks the higher potential of passive unloading.

## 4. Discussion

In this study, we explored the significance of the apoplastic to symplastic unloading switch and the transition from energy to storage sugar metabolism on tuber sink-strength increase during and after tuberization. We first investigated whether, in addition to the previously identified StSP6A-mediated decrease in apoplastic transport upon tuberization, also plasmodesmata further opened. For this, we used callose deposition as a proxy for plasmodesmata opening. Using fluorescence microscopy, we attempted to show that callose levels decreased in tuber samples; however, because of the presence of large amounts of starch, no robust quantitative conclusions could be drawn.

Therefore, we decided to find clues for a reduction of callose deposition by investigating gene expression changes of callose homeostasis genes, that is, CalS for synthesis and 1,3-BG for degradation. We observed that while 1,3-BGs from the γ-clade were expressed in tuber samples, expression of α- and β-clade is dominant in stolons. In Arabidopsis, the γ-clade proteins have been associated with pathogen resistance and cell wall remodelling (Doxey et al., 2007). Increased expression of the excreted γ-clade proteins might thus be an indicator of faster cell growth and has been associated with fast growth in pollen tubes (Wang et al., 2022). The majority of 1,3-BGs expressed in stolons are membrane- and/or PD-associated, suggesting a role in callose degradation. Expression patterns in stolons thus suggest high degradation potential at plasmodesmata, while in tubers this potential is decreased and growth-, and pathogen-associated expression dominates. Phylogenetic inference of function for the smaller CalS family revealed clear expression of gametophyte-associated proteins in the flower/stamen cluster (Soltu.DM.11G008010) and two putative PD-associated proteins (Soltu.DM.07G023050 and Soltu.DM.01G001920), which were highly expressed in fruit, callus and stolon samples. No to low expression of CalS in tubers was present. Combined, this analysis suggests that callose homeostasis at plasmodesmata is a constant process of synthesis and degradation in stolons, as also observed in *A. thaliana* pollen tubes, where callose is transiently present (Abercrombie et al., 2011). In contrast, in tubers, the transient presence of callose at plasmodesmata is replaced by low callose deposition due to decreased synthesis and relocation of callose degradation to the apoplastic space. It thus suggests that decreased callose deposition in tubers is caused by decreased synthetic CalS expression and not increased 1,3-BG expression. Recently, Nicolas et al. (2022) showed that both downregulation of CalS and upregulation of 1,3-BG resulted in increased symplastic movement in aerial tubers. There, the downregulation of CalS was again more prominent than the upregulation of 1,3-BG expression. Overall, these results show that next to the interaction of SWEET and SP6A at the protein level (Abelenda et al., 2019), also changes in callose and sucrose metabolism at the transcriptional level determine the change in unloading mode during tuberization.

We next investigated the concurrence of the changes in callose and sucrose metabolism. Expression of CalS and cwINV is exclusively clustered in stolon samples, whereas SuSy expression dominates in tubers. Low expression of SuSy genes was present in stolons, which can possibly be explained by the need for its product UDP-glucose in both stolons and tubers as it is a shared precursor for both starch and callose metabolism (Barnes & Anderson, 2018). Furthermore, low individual expression levels can be caused by technical or biological noise between the samples in this dataset. Overall, this indicates a well-coordinated developmental switch, with a combined transition from growth (cwInv) to storage (SuSy) metabolism and from callose PD homeostasis to extracellular callose degradation.

Finally, we set out to understand the implications of this coordinated switch on the unloading potential and thus sink strength of tubers. To this end, we parameterized a biophysics-based phloem unloading model (Ross-Elliot et al., 2017) for stolons and tubers. Using this model, we demonstrated that it is the combined switching of the unloading mode and the sucrose metabolism that increases tuber sink strength. The metabolic switch ensures maintenance of the concentration gradient necessary for efficient symplastic unloading, while starch metabolism is further activated because of increased cytoplasmic sucrose inflow (Stein & Granot, 2019; Winter & Huber, 2000). Clearly, the finding that passive gradient-driven transport increases with an enhanced gradient is in itself trivial. Additionally, the exact time point at which symplastic transport exceeds apoplastic transport in efficiency of course depends on the precise parameterization of apoplastic and symplastic transport rates and densities. The key point of our current modelling effort lies in the demonstration that – unless an unrealistic phloem concentration scenario is applied – during initial stolon and tuber development, apoplastic transport is more efficient, whereas at later stages, symplastic transport is more optimal. This underlines that 1) the *in planta* observed switch in the transport mode is physiologically sensible and 2) symplastic transport is not always more optimal. Indeed, in fruit plants, sucrose-storing fruits and seeds typically depend on a switch to apoplastic unloading to prevent a symplastic back-flow of the soluble sugars down the created concentration gradient (Ma et al., 2019). In the current model, enzyme levels were kept constant, as, for example, different SWEET subtypes showed different expression dynamics upon tuberization. Additionally, we kept enzyme activity constant, despite the previously demonstrated *StSP6A*-mediated decline in *StSWEET11* activity. Thus, in reality, active apoplastic transport would decrease in later stages in all scenarios, further favouring symplastic transport. On a similar note, we kept the PD density and area constant, while we showed that callose removal led to an increased openness and thus diameter. Other processes, such as increasing PD density over development or changes in other gating proteins, shown to increase PD conduction (Lucas et al., 2009) were also not considered. These changes would have further favoured symplastic over apoplastic unloading. Finally, we here used ‘simple’ PD architecture, shown to be up to 10x less effective than funnel plasmodesmata (Ross-Elliot et al., 2017). Overall, the model used here is thus a conservative, worst-case scenario. As such, the model strongly supports that it is the combined switching of sugar metabolism and unloading mode that enables an increased tuber sink strength.

Interestingly, tuberization onset requires the arrival of SP6Ain stolon parenchyma, and thus requires passage through plasmodesmata, implying that partial opening of plasmodesmata via callose removal must precede or coincide with SP6A arrival and tuberization. Indeed, Arabidopsis FT has been reported to dilate plasmodesmal channels to promote its own intercellular transport (Yoo et al., 2013), and possibly SP6A has a similar function. This would suggest an even further synchronisation of also the tuber developmental programme with unloading mode and metabolism, and would be an interesting direction for further research.

## Supporting information

van den Herik et al. supplementary material van den Herik et al. supplementary material

## Data Availability

No data were generated in this study.

## Supplementary material

The supplementary material for this article can be found at https://doi.org/10.1017/qpb.2024.4.
