# Supplementary material for: A coordinated switch in sucrose and callose metabolism enables enhanced symplastic unloading in potato tubers
Source: Quant Plant Biol. 2024 Apr 17;5:e4. doi: 10.1017/qpb.2024.4 (PMC11058582; doi:10.1017/qpb.2024.4)
Supplement: Peer review history

# Editor report: Recommendation: A coordinated switch in sucrose and callose metabolism enables enhanced symplastic unloading in potato tubers — R0/PR2

**DOI:** 10.1017/qpb.2024.4.pr2

**Affiliations:** School of Biological Sciences, University of Hong Kong, Hong Kong

Dear Authors

Thank you for submitting your manuscript to the QPB. We have received two referee reports from two reviewers. As you can see in their reports, there are concerns on the conclusion and the proposed model based on the existing data. Please revise the manuscript accordingly and prepare a point-to-point reply to the reviewers. The manuscript will only be accepted to the satisfaction of the reviewers. 

Yours sincerely

Boon Leong Lim

Associate Editor

QPB

PS. In addition to the reviewers' comments, I also spot some mistakes:

-Fig. 1. Legend. 

-Lots of typo errors: 

e.g. Phylogenetic, not ‘Phylogentic’ (Fig. S1, S2).

e.g. Arabidopsis, not ‘arabidopsis’ (Fig. S2).

-Use of capital letters in nomenclature. 

e.g. Beta-1,3-glucosidase (Fig. S1) and Callose Synthase (Fig. S2) should be in small cap. 

-Fig. S6. The gene accession numbers are too small to read. 

-The sources of the Kms of SWEET/SUC transporters should be cited (lines 313, 316). I think a Km of 50 mM is high rather than low. 

-How did you differentiate the signals from callose, lignin and starch in Fig. 1?

# Editor report: Decision: A coordinated switch in sucrose and callose metabolism enables enhanced symplastic unloading in potato tubers — R0/PR3

**DOI:** 10.1017/qpb.2024.4.pr3

**Affiliations:** RDP, ENS de Lyon, France

No accompanying comment.

# Editor report: Recommendation: A coordinated switch in sucrose and callose metabolism enables enhanced symplastic unloading in potato tubers — R1/PR5

**DOI:** 10.1017/qpb.2024.4.pr5

**Affiliations:** School of Biological Sciences, University of Hong Kong, Hong Kong

Dear Authors

Thank you for submitting a revised version. The reviewers still have some minor concerns. 

Can you please further improve the manuscript based on their comments? 

Reviewers' comments:

• Line 358: “much lower than the sucrose concentration in the leaves and stem, which can be up to 1.7M”. May I have the source of information?

I have doubt about the sucrose concentration in leaves and stem as 1.7M is extremely high. The M.W. of sucrose is 342 g/L. In orange juice the sucrose content is approximately 44g/L (0.128M) and in apple juice is approximately 13.2g/L (0.04M). The total sugar contents in orange juice and apple juice are approximately 90-130g/L. In the response letter the author wrote “we’ve previously simulated concentration profiles along the stem (van den Herik et al., 2021). This model indeed predicts high sucrose concentrations in the leaf phloem (up to 1.7M) and in the stem (1M to 1.2M)”. Can the author elaborate more on this in the manuscript?

• In the response letter there is a Table S3 and this table cited (van den Herik et al., 2020). However, I cannot find this article in the reference list of the manuscript and there is no supplementary table in the supplementary file. Please clarify. 

• Line 113 invertase

• Line 206 A space between (Fig. 1C,D) should be added.

• Full stops are missing in some Figure legends. 

• A full stop should be added after (Fig. S7) in line 383. 

• Fig S2. A. thaliana should be in italic. 

Yours sincerely

Boon Leong Lim

Associate Editor

QPB

# Editor report: Decision: A coordinated switch in sucrose and callose metabolism enables enhanced symplastic unloading in potato tubers — R1/PR6

**DOI:** 10.1017/qpb.2024.4.pr6

**Affiliations:** RDP, ENS de Lyon, France

No accompanying comment.

# Editor report: Recommendation: A coordinated switch in sucrose and callose metabolism enables enhanced symplastic unloading in potato tubers — R2/PR8

**DOI:** 10.1017/qpb.2024.4.pr8

**Affiliations:** School of Biological Sciences, University of Hong Kong, Hong Kong

Dear Authors

Thank you for your revision. 

In line 354, you wrote “For all three scenarios phloem concentrations...”

In line 358, you wrote " in line with experimental studies reporting values from 1.35M (Kehr et al., 

359 1998) to 1.8M (Pescod et al., 2007) in potato leaf phloem". 

In both lines ‘phloem’ was used. But in line 357 you wrote “ it predicted sucrose concentration in the leaves and stem up to 1.7M”. I think ‘leaves and stem’ are two tissues and are different from phloem. 

Should you use ‘phloem’ instead of 'leaves and stem" in line 357?

Yours sincerely

Boon Leong Lim

# Editor report: Decision: A coordinated switch in sucrose and callose metabolism enables enhanced symplastic unloading in potato tubers — R2/PR9

**DOI:** 10.1017/qpb.2024.4.pr9

**Affiliations:** RDP, ENS de Lyon, France

No accompanying comment.
